# Optimizing the “Time to pregnancy” in women with multiple sclerosis: the OPTIMUS Delphi survey

**DOI:** 10.3389/fneur.2023.1255496

**Published:** 2023-10-06

**Authors:** Luigi Carbone, Doriana Landi, Raffaella Di Girolamo, Paola Anserini, Diego Centonze, Girolama Alessandra Marfia, Carlo Alviggi, the Interdisciplinary Group for Fertility in Multiple Sclerosis (IGFMS), Carlo Alviggi, Raffaella Di Girolamo, Luigi Carbone, Roberta Lanzillo, Doriana Landi, Girolama Alessandra Marfia, Paola Anserini, Diego Centonze, Pietro Annovazzi, Simona Bonavita, Giovanna Borriello, Paola Cavalla, Raffaella Cerqua, Marinella Clerico, Eleonora Cocco, Cinzia Cordioli, Emanuele D’Amico, Giovanna De Luca, Massimiliano Di Filippo, Roberta Fantozzi, Diana Ferraro, Pietro Iaffaldano, Matilde Inglese, Paola Perini, Emilio Portaccio, Paolo Ragonese, Valentina Torri Clerici, Carla Tortorella, Paola Valentino

**Affiliations:** ^1^Department of Neuroscience, Reproductive Sciences and Dentistry, School of Medicine, University of Naples Federico II, Naples, Italy; ^2^Multiple Sclerosis Clinical and Research Unit, University Hospital of Rome Tor Vergata, Rome, Italy; ^3^Department of Public Health, School of Medicine, University of Naples Federico II, Naples, Italy; ^4^UOS Physiopathology of Human Reproduction, IRCCS Ospedale Policlinico San Martino, Genova, Italy; ^5^Department of Systems Medicine, Laboratory of Synaptic Immunopathology, “Tor Vergata” University, Rome, Italy; ^6^Unit of Neurology, IRCCS Neuromed, Pozzilli, Isernia, Italy

**Keywords:** multiple sclerosis, infertility, time to pregnancy, Delphi, assisted reproductive technology

## Abstract

**Background:**

The debate on how to manage women affected by multiple sclerosis (MS) during reproductive age is still open, as is the issue of fertility in such patients. Main issue regard the identification of the optimal window for pregnancy and how to deal with medical therapy before and during conception. The aim of this Delphi consensus was to collect the opinions of a multidisciplinary group, involving reproductive medicine specialists and neurologists with experience in the management of multiple sclerosis women with reproductive desire.

**Methods:**

Four experts plus scientific coordinators developed a questionnaire distributed online to 10 neurologists and later discussed the responses and amended a list of statements. The statements were then distributed *via* an online survey to 23 neurologists (comprising the first 10), who voted on their level of agreement/disagreement with each statement. Consensus was achieved if agreement or disagreement with a statement exceeded 66%.

**Results:**

Twenty-one statements reached consensus after two rounds of voting, leading to the following main recommendations: (1) Fertility evaluation should be suggested to wMS, in case of the need to shorten time to pregnancy and before treatment switch in women on DMTs contraindicated in pregnancy, particularly in case of highly active disease and age > 35 years. (2) ART should not be discouraged in wMS, but the use of DMTs until pregnancy confirmation should be suggested; ART may be considered in order to reduce time to pregnancy in MS women with a reduced ovarian reserve and/or age > 35 years, but in case of an expected poor ART prognosis and the need for more than one ART cycle, a switch to a high-efficacy DMD before ART should be offered. (3) Oocyte cryopreservation may be considered in women with reduced ovarian reserve, with unpredictable time to complete diagnostic workup and achieve disease control; a risk/cost–benefit analysis must be performed in women >35 years, considering the diminished ovarian reserve.

**Conclusion:**

This consensus will help MS neurologists to support family planning in wMS, respecting MS therapeutic needs while also taking into account the safety and impact of advancing age on fertility.

## 1. Introduction

Multiple sclerosis (MS) is an autoimmune demyelinating disease of the central nervous system (CNS) with a female-to-male sex ratio of 3:1 (1). It is more common among women of reproductive age (2). Previously, MS was considered an obstacle for motherhood, given the huge impact that the disease had on the quality of life of affected women, the social stigma, and the lack of data on fetal outcomes (3). Although the rate of childlessness is still higher in MS women compared to the general population, it is now ascertained that pregnancy in MS women is not associated with adverse obstetric outcomes (4). Likewise, MS course does not worsen during pregnancy (5). However, an increased risk of relapse after pregnancy is reported, especially in women with MS who relapse shortly before pregnancy and with higher pre-conceptional disability (6). Currently, the issue of fertility in women with MS is still debated. Roux et al. observed that the fecundity of MS women seems not different from the general population (7). However, some epidemiological studies have shown that women with MS may have fewer children than the general population (8). To date, the improvements in the clinical conditions of patients, obtained through the use of increasingly effective disease-modifying drugs (DMDs), have favored the openness to the maternity project by both patients and neurologists (9). It remains to be clarified, however, whether MS causes a reduction in fertility, as occasionally reported in the literature, and whether this condition should be ascribed to the therapies or to the disease itself (10–14). Potential underlying reasons for this could include the effects of the autoimmune disease on fertility (15). Furthermore, taking into account the ever-increasing age at which women are planning pregnancy nowadays, it happens more and more often that women with MS find themselves in the need to request assisted reproductive technology (ART) treatments to achieve pregnancy (16). In light of this scenario, it appears of striking importance to define strategies to manage women with MS who express a desire for conception, whether it can occur spontaneously or requires access to ART programs, with the aim of encouraging the realization of the maternity project in a context of safeguarding the neurological health of women with MS. As pregnancy planning is a fundamental driving factor in the treatment decision-making of women with MS, there is an emerging need to define how and when the fertility of the women with MS and more generally of the couple should be assessed to provide accurate and up-to-date counseling. The aims of this Delphi consensus were (1) to collect the expert opinion of neurologists involved in MS treatment and with expertise in pregnancy management, about the best practice in handling the reproductive desire in MS women and treatment plan in relation to pregnancy planning, and (2) to address the issue of couples’ fertility evaluation and the feasibility of ART treatments and oocyte preservation, in collaboration with reproductive medicine experts, with the purpose to optimize the time to pregnancy while minimizing the risk of relapses and undertreatment in MS women.

## 2. Materials and methods

### 2.1. Participants

The Delphi consensus involved a scientific board, comprising the scientific coordinators (CA, DC, and GAM) and four additional experts (PA, DL, LC, and RDG). A panel of 10 experts and, successively, an extended panel of 23 experts (comprising the first 10) were suggested by the scientific board. The panel comprised neurology experts working in the field of MS (Table 1). Experts have been selected according to the following criteria: (1) clinical/research experience on the topic of pregnancy/fertility/women’s health in MS and/or (2) consolidated experience in the management of wMS (i.e., working in large Italian MS centers). Geographical provenance has been considered in order to ensure that representatives from all the main Italian regions are included.

**Table 1:** Participants involved in the Delphi consensus process.

Name	Place	Step 1 Questionnaire development	Step 2 Questionnaire distribution	Step 3 Statements’ development	Step 4 Statements’ grading	Step 5 Statements’ Rephrasing	Step 6 Statements’ grading
Carlo Alviggi*	Naples	Y		Y		Y	
Diego Centonze*	Rome	Y		Y		Y	
Gerola Alessandra Marfia*	Rome	Y		Y		Y	
Paola Anserini*	Genova	Y		Y		Y	
Doriana Landi*	Rome	Y		Y		Y	
Luigi Carbone*	Naples	Y		Y		Y	
Raffaella Di Girolamo*	Naples			Y		Y	
Eleonora Cocco	Cagliari		Y		Y		Y
Emilio Portaccio	Florence		Y		Y		Y
Roberta Lanzillo	Naples		Y		Y		Y
Simona Bonavita	Naples		Y		Y		
Paola Perini	Padua		Y		Y		Y
Diana Ferraro	Modena		Y		Y		Y
Matilde Inglese	Genova		Y		Y		Y
Marinella Clerico	Turin		Y		Y		Y
Emanuele D’Amico	Catania		Y		Y		Y
Pietro Annovazzi	Gallarate		Y		Y		Y
Carla Tortorella	Rome				Y		Y
Giovanna Borriello	Rome				Y		Y
Massimiliano Di Filippo	Perugia				Y		Y
Paola Cavalla	Turin				Y		Y
Raffaella Cerqua	Ancona				Y		Y
Giovanna De Luca	Chieti				Y		
Roberta Fantozzi	Pozzilli				Y		Y
Paola Valentino	Catanzaro				Y		
Paolo Ragonese	Palermo				Y		Y
Pietro Iaffaldano	Bari				Y		
Cinzia Cordioli	Brescia				Y		Y
Valentina Torri Clerici	Milan				Y		Y
Cinzia Scandellari	Bologna				Y		Y

*Scientific board members.

### 2.2. The consensus process

The scientific board generated a questionnaire (Step 1) with the aim of identifying the key topics and gaps in the treatment of fertility in women with MS. The questionnaire included open and multiple-choice questions and was distributed online to a restricted panel of 10 experts (Step 2). Based on the replies, the scientific coordinators developed the initial statements (Step 3). In Step 3, the scientific board discussed these statements during two web conferences, having the possibility to add, remove, or amend the proposed statements and references. The final selection of statements was decided by consensus and approved by the scientific coordinator and scientific board by email.

**Figure 1:**
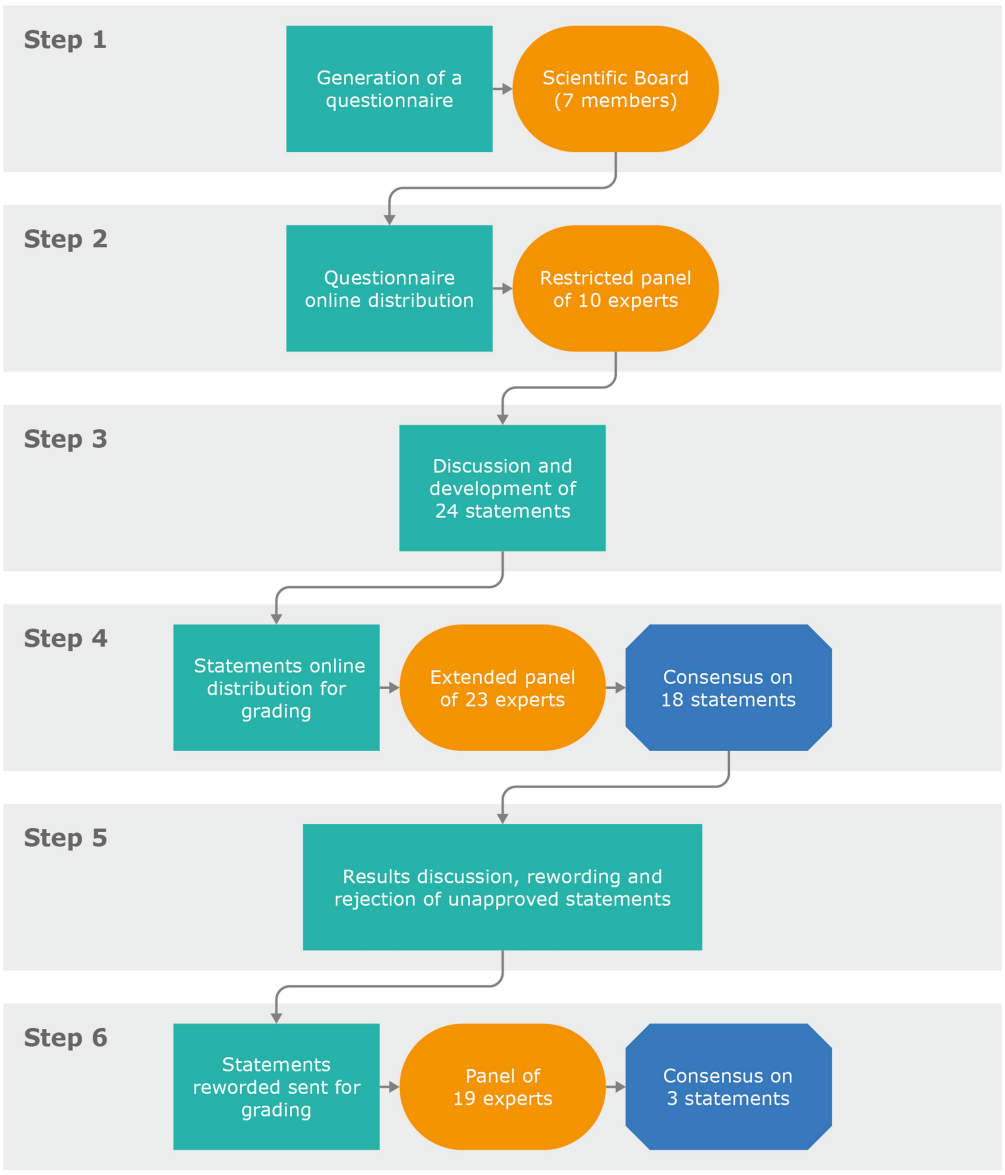
Steps of the Delphi consensus process.

In Step 4, an online survey of the statements was circulated to the extended panel. Each participant anonymously rated his/her level of agreement with each statement using a 5-item Likert scale: 1 = totally disagree; 2 = disagree; 3 = neither agree nor disagree; 4 = agree; 5 = totally agree. Participants were also asked to provide the main reasons for their chosen level of agreement or disagreement (free text). Consensus was considered to have been achieved if the proportion of participants either disagreeing with a statement (responding 1 or 2) or agreeing with a statement (responding 4 or 5) exceeded 66%. If the proportion of participants either agreeing or disagreeing with a statement did not exceed 66%, that statement was discussed according to the feedback received and rephrased. In Step 5, the results of the online survey were discussed in a web conference by the scientific board. Another survey, including only the rephrased statement(s), was sent for a further round of voting (Step 6). The protocol required that this process be repeated, with the statements being revised, until consensus was reached for every statement (Figure 1).

## 3. Results

### 3.1. Results overview

**Figure 2:**
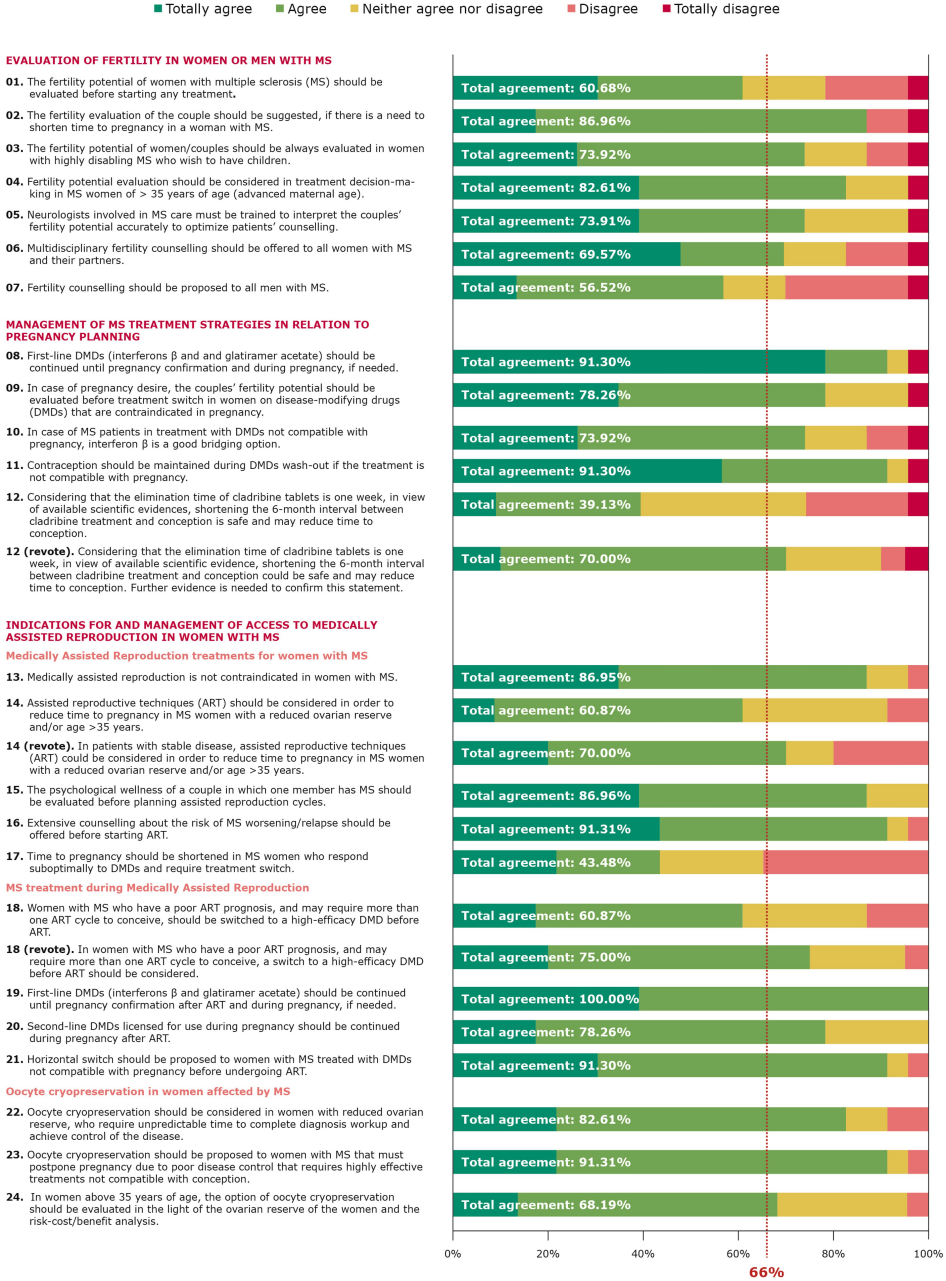
Grading of the statements.

The scientific board developed 24 statements (Table 2). Consensus on each statement was reached in a web conference and subsequent email discussion. All members of the scientific board approved the final wording. The 24 statements approved by the scientific board were related to the evaluation of fertility in women/couples with MS (7 statements); management of MS treatment strategies in relation to pregnancy planning (5 statements); and indications for and management of access to medically assisted reproduction in women with MS (12 statements), divided into three subsections: medically assisted reproduction treatments for women with MS, MS treatment during medically assisted reproduction, oocyte cryopreservation in women affected by MS. Overall, 23 members of the extended panel completed the entire survey. All statements were rated by all experts. Consensus was achieved for 18 of the 24 statements after the first round of voting. Statements that did not reach the consensus threshold were statements 1, 7, 12, 14, 17, and 18. For three statements (12, 14, and 18) for which consensus threshold was not achieved, the neutral option (neither agree nor disagree) was higher than the sum of the “disagree” option. The comments provided are shown in the Supplementary material. The mean neutral opinion on the first round was 14% (max 34.78%; min 0%). Considering only the statements that reached the consensus in the first round, the mean agreement was 83% (max 100%; min 68%; Figure 2). Following a discussion concerning the statements that did not reach an agreement, the scientific board decided to reject three of them (1, 7, 17) for the following reasons: Statements 1 and 7 concerned fertility evaluation of all men and women with MS, regardless desires, conditions, etc. The board agreed that the evaluation of fertility potential, based on patients’ conditions and desires, was well-covered by the other statements under the fertility evaluation section. Statement 17 was considered redundant since the time to pregnancy and treatment switch are discussed in greater detail in other statements of the section. Three statements (12, 14, and 18) were rephrased and circulated to the panel for a second round of voting (Step 6). Nineteen out of 23 experts participated in the second round. All the statements rephrased reached consensus. Statements are discussed in detail below. To conclude, experts agreed on three main concepts that arose from this consensus (Table 3).

**Table 2:** Statements approved by the scientific board.

	Evaluation of fertility in women or men with MS
1.	The fertility potential of women with multiple sclerosis (MS) should be evaluated before starting any treatment.
2.	The fertility evaluation of the couple should be suggested, if there is a need to shorten the time to pregnancy in a woman with MS.
3.	The fertility potential of women/couples should be always evaluated in women with highly active MS who wish to have children.
4.	Fertility potential evaluation should be considered in treatment decision-making in MS women of >35 years of age (advanced maternal age).
5.	Neurologists involved in MS care must be trained to interpret the couples’ fertility potential accurately to optimize patients’ counseling.
6.	Multidisciplinary fertility counseling should be offered to all women with MS and their partners.
7.	Fertility counseling should be proposed for all men with MS.
	Management of MS treatment strategies in relation to pregnancy planning
8.	First-line DMDs (interferons β and glatiramer acetate) should be continued until pregnancy confirmation and during pregnancy, if needed.
9.	In case of pregnancy desire, the couples’ fertility potential should be evaluated before treatment switch in women on disease-modifying drugs (DMDs) that are contraindicated in pregnancy.
10.	In case of MS patients in treatment with DMDs not compatible with pregnancy, interferon β is a good bridging option.
11.	Contraception should be maintained during DMD washout if the treatment is not compatible with pregnancy.
12.	Considering that the elimination time of cladribine tablets is 1 week, in view of available scientific evidence, shortening the 6-month interval between cladribine treatment and conception is safe and may reduce the time to conception.
12 revote.	Considering that the elimination time of cladribine tablets is 1 week, in view of available scientific evidence, shortening the 6-month interval between cladribine treatment and conception could be safe and may reduce the time to conception. Further evidence is needed to confirm this statement.
	Indications for and management of access to medically assisted reproduction in women with MS
	Medically assisted reproduction treatments for women with MS
13.	Medically assisted reproduction is not contraindicated in women with MS.
14.	Assisted reproductive techniques (ARTs) should be considered in order to reduce time to pregnancy in MS women with a reduced ovarian reserve and/or age > 35 years.
14 revote.	In patients with stable disease, assisted reproductive techniques (ARTs) could be considered in order to reduce time to pregnancy in MS women with a reduced ovarian reserve and/or age > 35 years.
15.	The psychological wellness of a couple in which one member has MS should be evaluated before planning assisted reproduction cycles.
16.	Extensive counseling about the risk of MS worsening/relapse should be offered before starting ART.
17.	Time to pregnancy should be shortened in MS women who respond suboptimally to DMDs and require treatment switch.
	MS treatment during medically assisted reproduction
18.	Women with MS who have a poor ART prognosis, and may require more than one ART cycle to conceive, should be switched to a high-efficacy DMD before ART.
18 revote.	In women with MS who have a poor ART prognosis, and may require more than one ART cycle to conceive, a switch to a high-efficacy DMD before ART should be considered.
19.	First-line DMDs (interferons β and glatiramer acetate) should be continued until pregnancy confirmation after ART and during pregnancy, if needed.
20.	Second-line DMDs licensed for use during pregnancy should be continued during pregnancy after ART.
21.	Horizontal switch should be proposed for women with MS treated with DMDs not compatible with pregnancy before undergoing ART.
	Oocyte cryopreservation in women affected by MS
22.	Oocyte cryopreservation should be considered in women with reduced ovarian reserve, who require unpredictable time to complete diagnosis workup and achieve control of the disease.
23.	Oocyte cryopreservation should be proposed to women with MS who must postpone pregnancy due to poor disease control that requires highly effective treatments not compatible with conception.
24.	In women above 35 years of age, the option of oocyte cryopreservation should be evaluated in light of the ovarian reserve of the women and the risk-cost/benefit analysis.

**Table 3:** Three main concepts of the survey.

Fertility evaluation should be suggested to wMS, in case of need to shorten time to pregnancy and before treatment switch in women on DMTs contraindicated in pregnancy, particularly in case of highly active disease and age > 35 years.	ART should not be discouraged in wMS, but use of DMTs until pregnancy confirmation should be suggested; ART may be considered in order to reduce time to pregnancy in MS women with a reduced ovarian reserve and/or age > 35 years, but in case of expected poor ART prognosis and need of more than one ART cycle, a switch to a high-efficacy DMD before ART should be offered.	Oocyte cryopreservation may be considered in women with reduced ovarian reserve, with unpredictable time to complete diagnostic workup and achieve disease control; a risk/cost–benefit analysis must be performed in women >35 years considering the diminished ovarian reserve.

### 3.2. Multiple sclerosis statements approved (first and second rounds)

#### 3.2.1. Topic 1. Evaluation of fertility in women or men with MS

S2. The fertility evaluation of the couple should be suggested, if there is a need to shorten time to pregnancy in a woman with MS.

S3. The fertility potential of women/couples should be always evaluated in women with highly disabling MS who wish to have children.

S4. Fertility potential evaluation should be considered in treatment decision-making in MS women of >35 years of age (advanced maternal age).

S5. Neurologists involved in MS care must be trained to interpret the couples’ fertility potential accurately to optimize patients’ counseling.

S6. Multidisciplinary fertility counseling should be offered to all women with MS and their partners.

The statements concerning fertility evaluation reached 86.96, 73.92, 82.61, 73.91, and 69.57% agreement, respectively. In women affected by MS, shortening the time to pregnancy could depend both on fertility and MS issues. Given that no biomarkers exist to discriminate fecundity, time to pregnancy is becoming a useful surrogate to describe it at the population level (17, 18). In fact, in relation to fertility issues, it is well acknowledged that fertility is a time-dependent condition that declines with aging (19). The decline in female fertility is constant after 30 years of age, but increases dramatically after 35 years (20). In this regard, both the quantity and the quality of oocytes decrease, with an increase in miscarriage rates and aneuploidy (20–22). Indeed, ovarian reserve markers reflect the pool of oocytes a woman might benefit for reproductive purposes and time to pregnancy shortens with increasing levels of them (23). Furthermore, advanced maternal age (i.e., over 35 years old) has been associated with an increase in adverse pregnancy outcomes (24, 25). Therefore, time to pregnancy acquires even more importance in relation to chronic and autoimmune diseases, whose incidence usually peaks during reproductive years and where various issues coexist, determining reproductive concerns. Specifically, fertility issues in MS have been a matter of debate for years (26). Despite reports of an increased prevalence of infertility among MS women (27, 28), the heterogeneity of the data and the presence of numerous confounding factors have not yet allowed for definitive conclusions (29). In particular, although pregnancy for MS women does not seem associated with severely adverse obstetric outcomes (30), the higher rate of childlessness among MS patients (31) could be explained by psychosocial factors, such as current disability or fear of future problems, fear of genetically transmitting MS, fear of not starting/discontinuing treatments (32), coexisting with biological factors, such as sexual dysfunction, reduced libido, altered sensitivity, abnormal endocrine patterns, and ovarian reserve (26). In addition, ovarian reserve has been studied in MS women with contrasting results (10–14, 33, 34). Interestingly, when the disease course is worse, the ovarian reserve has been found to be lower (12, 33), thereby complicating the eventual reproductive project. Moreover, considering different clinical phenotypes and the difficulty in predicting disease progression (35), fertility evaluation remains a remarkable practice to carry out in MS women who wish to have children whatsoever, even when there is a high activity of disease, the resolution of which could take years and thus be associated with a reduced ovarian reserve. Concomitantly, women with higher disease activity may require treatment with more powerful drugs; currently, there are very few data about the safety of the fetus for the majority of these treatments. For these reasons, women with worse prognostic factors desiring pregnancy may choose to be undertreated while trying to conceive or to waive the reproductive project. In this population, an objective assessment of fertility could be of utmost importance as it may dramatically impact patients’ counseling in order to limit avoidable disability or to influence treatment choices. Considering the age-related decline of fertility, its evaluation should be explicitly proposed to women >35 years old, since later it may be too late. On the other side, it should not be taken for granted that women with MS aged >35 years should waive pregnancy desire. Bonavita et al. observed that childlessness was more common in the subgroup of patients aged 36–45 years (8), and in 78% of cases, the treatment was not selected considering family planning. In the general population, the average age of women at the birth of their first child has increased (36) for several social reasons that are also shared by women with MS. Additionally, these women start pregnancy planning after having spent years trying to achieve disease remission. Moreover, access to ART programs is becoming more common among healthy women as well as women with MS. Therefore, fertility evaluation should become ordinary practice at least in women >35 years before making treatment choices to individually tailor counseling and preserve both women’s health and pregnancy plans. Thus, medical counseling acquires the utmost significance in driving women’s and couple’s choices regarding family planning. In 2014, Wundes et al. (37) evaluated what healthcare providers say to MS women about pregnancy, showing that almost all surveyed participants did not discourage pregnancy based solely on MS diagnosis, but few encouraged it, and a hypothesis could be that the lack of active encouragement might be perceived as a lack of support. Indeed, nowadays, such an attitude seems outdated. In addition, recommendations about DMD use vary considerably (37, 38). Recently, various attempts have been made to resume the main counseling issues and related management options for MS women with reproductive desires (39–43). Currently, the main topic of discussion is the therapeutic management of the window between pregnancy desire and conception as well as during gestation (44, 45). However, the issue of a couple’s fertility evaluation before making such choices has never been addressed. Nevertheless, it should also be taken into consideration the partner’s role in reproductive issues. As per definition, “infertility is a disease of the male or female reproductive system defined by the failure to achieve a pregnancy after 12 months or more of regular unprotected sexual intercourse” (46). Therefore, although not mandatory, it seems advisable that both partners be evaluated whenever planning a pregnancy, in order to assess whether infertility issues could complicate the road to conception. Initial screenings might include semen analysis and ovarian reserve markers’ evaluation, as well as questions about menstrual cycle regularity, whose first interpretation could be done by a neurologist. Moreover, creating a multidisciplinary team of reproductive medicine experts would speed up the process. Then, this would help the MS treating physicians manage therapeutic options. These statements received 13.04, 26.08, 17.39, 26.09, and 30.43% disagreement or doubt (neither agree nor disagree), respectively. The motivations supporting disagreements are outlined in the Supplementary material. Of note, respondents argued to consider age, the severity of the disease, and true desire for motherhood as determinants in deciding fertility evaluation.

#### 3.2.2. Topic 2. Management of MS treatment strategies in relation to pregnancy planning

S8. First-line DMDs (interferons β and glatiramer acetate) should be continued until pregnancy confirmation and during pregnancy, if needed.

S9. In case of pregnancy desire, the couples’ fertility potential should be evaluated before treatment switch in women on disease-modifying drugs (DMDs) that are contraindicated in pregnancy.

S10. In case of MS patients in treatment with DMDs not compatible with pregnancy, interferon β is a good bridging option.

S11. Contraception should be maintained during DMDs wash-out if the treatment is not compatible with pregnancy.

S12 (rephrased). Considering that the elimination time of cladribine tablets is 1 week, in view of available scientific evidence, shortening the 6-month interval between cladribine treatment and conception could be safe and may reduce time to conception. Further evidence is needed to confirm this statement.

The statements concerning MS treatment decision-making reached 91.3, 78.26, 73.92, 91.3, and 70.0% agreement, respectively. It is now well-established that pregnancy reduces the risk of relapses in MS women (5, 30) with pregnancy advancement, due to the hormonal-driven downregulation of proinflammatory immune mechanisms. Despite robust evidence regarding the favorable course of the disease in pregnancy, there is still a longstanding debate about whether DMDs should be suspended before conception and for how long, considering that today the majority of women with MS of childbearing age are treated with these medications. Main concerns regard the safety of the exposure of the fetus to DMDs at the time of conception or during pregnancy; for this reason, in the past, DMDs were usually discontinued in women planning pregnancy before trying to conceive. Nevertheless, several lines of evidence identified, among other factors, the length of treatment washout before conception as a predictor of a higher risk of relapse (47–49). Therefore, the last ECTRIMS/EAN MS treatment guidelines (50) recommended considering continuing interferon (IFN) or glatiramer acetate until pregnancy is confirmed in women with a high risk of relapses, also keeping in mind that often it is not possible to predict the time to pregnancy. Injectables, in fact, are now considered safe for the fetus and are labeled for use during pregnancy and lactation (51–56). While the oldest studies showed that exposure to IFN-beta was associated with an increased risk of lower mean birth weight, shorter mean birth length, and preterm birth (57, 58), more recent works on large pregnancy registries disconfirmed these results and demonstrated that IFN-beta exposure before conception and/or during pregnancy does not adversely increase the rate of congenital anomalies or spontaneous abortions (59–61). Fewer studies have investigated the outcomes of infants exposed to glatiramer acetate showing that the exposure during the first trimester does not affect perinatal outcomes (62, 63). Limited data are available on the risk/benefit of continuing injectables DMD throughout pregnancy (64, 65), and new longitudinal studies are needed to address this issue. However, in agreement with ECTRIMS guidelines (50), such an option should be discussed with future parents, particularly in case of women with pre-conceptional adverse prognostic factors. A discussion is also open on the management of women seeking pregnancy that are treated with DMDs different from IFNs and glatiramer acetate. All of them are not licensed during pregnancy. Therefore, women who are on treatments not allowed during pregnancy should be ideally switched to other treatment options or discontinue therapies before conception. However, discontinuation of therapies may increase the risk of relapses and disability progression (66, 67). Several studies have shown that the odd of MS rebound after suspension, also motivated by pregnancy reasons, is particularly higher in women treated with sequestering drugs (i.e., natalizumab and fingolimod) (68–71), due to the rapid immune reconstitution occurring after treatment interruption. Hence, in this population, it would be advisable to assess women and couples’ fertility before the treatment switch, also considering couples’ age and the unknown toxic effect of chronic inflammation or DMDs (72) on ovarian reserve. Eventual detection of infertility before the treatment switch, which is not rare in older women, might help to optimize the timing of sequencing, in order to limit the risk of disability on one hand and to precociously manage treatable causes of infertility. Krysko et al. suggested (73) that, although not licensed for use during pregnancy, natalizumab could be continued at extended intervals dosing up until the third trimester, when it should be stopped to not incur in neonatal cytopenia (74). In addition, they proposed to target the last dose of anti-CD20 shortly before pregnancy, but ideally not during it. However, they have not stated the management during ART procedures, but underlined that women approaching fertility treatments should be on optimal disease control with a pregnancy-compatible DMD. Instead, in women with low-moderate disease activity treated with platform therapies not compatible with pregnancy and thus requiring lateral treatment switch, IFNs are considered a good bridging option due to the low risk of fetal abnormalities and the possibility of continuing treatments during pregnancy. In this case, it should be taken into account that the estimated therapeutic lag for relapses from treatment start ranges from 14 to 19 weeks (75); therefore, particularly for those women getting pregnant soon after treatment starts, it might be proposed to continue treatment during pregnancy to optimize treatment benefit. Conversely, contraception is recommended for all women on DMDs not compatible with pregnancy. Contraceptive methods are safe in MS; highly effective methods, including IUDs (intra-uterine devices) and implants, should be proposed to women taking DMDs with known teratogenicity (6, 76), and they should be maintained during treatment washout. Such an interval depends on the elimination half-life of each DMD; in fact, a drug is considered to have been fully eliminated after five half-lives. Currently, drug plasmatic concentration can be dosed only for teriflunomide, allowing personalizing of discontinuation of contraception when a plasma level of <0.02 mg/L is detected (77). For all the other DMDs, label indications should be followed. Nevertheless, for some of the most recently approved drugs, such as cladribine or anti-CD20 drugs (78), the recommended washout interval before trying to conceive exceeds the presumed drug half-life. For these treatments, regulatory authorities adopted a precautionary approach considering the lack of data on fetal safety and the prolonged immunological effects of these treatments, despite their low frequency of administration. In the case of cladribine tablets, the complete drug elimination time is 1 week (79), while it is recommended that women prevent pregnancy during treatment and for at least 6 months after the last dose. Moreover, as it is not known if cladribine may reduce the efficacy of birth control pills, a barrier method of contraception should be added during treatment and for at least 4 weeks after the last dose. Considering its pharmacodynamic and pharmacokinetic, shortening the 6-month interval between cladribine treatment and conception seems safe and might represent a promising approach to reducing time to conception in women treated with this drug, in particular in those >35 years old. These statements received 8.7, 21.74, 26.08, 8.7, and 30% disagreement or doubt (neither agree nor disagree), respectively. The motivations supporting these disagreements are outlined in the Supplementary material. In detail, experts debated whether glatiramer acetate is a good bridging option; also, it would be better to gain more evidence before considering it safe to conceive prior to 6 months after cladribine treatment and to consider the effects on white blood cell count.

#### 3.2.3. Topic 3. Medically assisted reproduction treatments for women with MS

S13. Medically assisted reproduction is not contraindicated in women with MS.

S14 (rephrased). In patients with stable disease, assisted reproductive techniques (ART) could be considered in order to reduce time to pregnancy in MS women with a reduced ovarian reserve and/or age > 35 years.

S15. The psychological wellness of a couple in which one member has MS should be evaluated before planning assisted reproduction cycles.

S16. Extensive counseling about the risk of MS worsening/relapse should be offered before starting ART.

The statements concerning ART in wMS reached 86.95, 70, 86.96, and 91.31% agreement, respectively. The safety of ART in MS women is still debated, as evidence reports an increased risk of disease reactivation after such procedures, which is mainly related to sex hormone manipulation and its proinflammatory effects (80–84). Not only clinical but also radiological evidence of relapse has been observed (85, 86). Currently, ART protocols for ovarian stimulation involve the use of GnRH agonists or antagonists to avoid the LH (luteinizing hormone) surge and the risk of spontaneous ovulation. While some studies showed increased relapse risk after GnRH agonist use (82–84), others did not find the same evidence (80, 81, 87); however, a recent meta-analysis demonstrated that no difference in risk of MS relapse was found between GnRH agonist and antagonist ART protocols (88). In addition, it has been observed that only wMS patients who suffered from relapses close to the start of the ART procedure were at risk of further relapses (89). Bove et al. (88) explored any confounding factors and found that age, parity, multiple IVF attempts, time without MS drugs, and disease duration had no effect on the association between ART and increased risk of relapse; interestingly, they also noticed that miscarriage after ART increased the risk of relapse 3 months after ART compared to 3 months before. The largest available study evaluating the relapse rate in 225 wMS undergoing ART compared 3-month exposed periods after IVF with unexposed periods before IVF and did not evidence an increase in the risk of relapse. Moreover, the results of this study do not support the hypothesis that patients stimulated with the GnRH agonist protocol have an increased risk of relapse (90). Finally, it was observed that there is no difference in live birth rates after ART between wMS and women without MS (16, 27). A very recent multicenter retrospective analysis observed that the relapse rate was not increased after ART and that being on therapeutic DMD was associated with a reduced relapse rate 3 months after ovarian stimulation: 10 out of the 13 patients relapsing after ART (over a 12-month period) were not on DMDs (91), enhancing the importance of continuing therapy when ART procedures are planned and performed.

Therefore, taking into account these data, it can be affirmed that MS is not a contraindication to ART treatments per sè, but a correct framework of the clinical conditions of the patients should be performed to allow its planning in the optimal window both for MS and pregnancy prognosis.

The diagnosis of infertility is associated with increased levels of emotional distress, anxiety, and depression (92). The importance of a couple’s psychological evaluation and management was also recognized by the European Society of Human Reproduction and Embryology (ESHRE); in fact, Gameiro et al. (93) released a guideline on how to manage the main psychological aspects that could arise before, during, and after treatment for couples seeking fertility treatments. To overcome this issue, it was declared that psychological issues should be assessed and care should be tailored, especially in cases of ART failure. In this regard, specific tools could also be used (94). Actually, MS adds extra stress to the already acknowledged psychological burden with which couples are requested to deal when they need to refer to assisted reproduction in order to conceive. All the worries declared by people with MS, such as fear of disability, fear of transmitting the disease, fear of not being able to care for children, and fear of discontinuing MS treatments, would probably be the main reasons that could affect the decision not only to become pregnant but also to access ART programs in cases of infertility diagnosis (8, 31, 32). Actually, Houtchens et al. (27) observed that, despite the fact that a higher proportion of wMS have been found infertile compared to healthy controls, less wMS seek infertility treatments than women without MS. In contrast, Sadovnick et al. (38) observed that the proportion of MS women requesting ART treatments was not different from the general population. This data could be read in the way that infertile wMS are scared by the additional stressful path of assisted reproduction to be added to their chronic condition, thereby giving up on the idea of family planning. This is why multidisciplinary and extensive counseling is strongly needed for infertile wMS approaching the possibility of ART treatments to conceive, to help them freely make reproductive choices after a thorough discussion on all the aspects, from the psychological to the pharmacological and clinical ones, covering the periods from before to after ART treatments and pregnancy. Indeed, wMS and their partners should be aware of the possibly increased risk of relapse after such treatments, which should be adequately discussed in place of pre-ART counseling by both the neurologist and the reproductive medicine specialist in relation to the abovementioned evidence and all the possible interfering factors. It has already been suggested that would be wiser to perform ART treatments during periods of disease stability (95, 96). Main indications remain related to infertility causes, but in order to not increase the likelihood of failure, it seems reasonable to consider wMS with reduced ovarian reserve and over 35 years old as the ones with time-dependent infertility conditions and therefore to get them soon into ART treatments. These statements received 13.05, 30, 13.04, and 8.69% disagreement or doubt (neither agree nor disagree), respectively. The motivations supporting these disagreements are outlined in the Supplementary material. Experts commented on the importance of the IVF protocol but also the need to inform patients about the risk of disease reactivation.

#### 3.2.4. Topic 4. MS treatment during medically assisted reproduction

S18 (rephrased). In women with MS who have a poor ART prognosis, and may require more than one ART cycle to conceive, a switch to a high-efficacy DMD before ART should be considered.

S19. First-line DMDs (interferons β and glatiramer acetate) should be continued until pregnancy confirmation after ART and during pregnancy, if needed.

S20. Second-line DMDs licensed for use during pregnancy should be continued during pregnancy after ART.

S21. Horizontal switch should be proposed to women with MS treated with DMDs not compatible with pregnancy before undergoing ART.

The statements concerning MS treatments during ART procedures in wMS reached 75, 100, 78.26, and 91.3% agreement, respectively. Taking into account that it is still debated if MS or eventually some MS drugs could have an impact on fertility, whenever a wMS should ask for ART treatments to achieve a pregnancy, it seems appropriate to evaluate her (assisted) reproductive prognosis in advance, to tailor the optimal reproductive strategy. Indeed, the best parameters to define the prognosis of ART procedures include the combination of age, ovarian reserve markers such as antral follicle count (AFC) and Anti-Müllerian hormone (AMH), as well as the number of oocytes retrieved in previous IVF cycles. Embryo euploidy also has a fundamental role in the achievement of pregnancy, and its relationship with age should be taken into account, given that its probability decreases with increasing maternal age (97–100), for which pre-implantation tests can be performed (101, 102). Therefore, in women with poor ART prognosis, an accumulation strategy could be proposed in order to increase the number of mature oocytes that would reasonably allow for at least one euploid embryo (103–106). Actually, a recent large cohort study based on nationwide Danish health registries analyzing 2,267 embryo transfers in 815 women with MS has shown that the chance of a live birth was not decreased in these women compared with women without MS undergoing ART (16). Nevertheless, it should be considered that wMS could undergo biochemical pregnancy, miscarriage (107), repeated implantation failure, and therefore, several embryo transfer attempts. These unfavorable ART events might multiply the risk of disease reactivation, for the abovementioned reasons, much more than in case of ART success (92).

Therefore, in women with unfavorable prognostic factors regarding ART and/or MS, it is advisable to switch to higher efficacy DMDs before ART. Accumulating evidence suggests that natalizumab or anti-CD20 drugs can be viable options, as they have no impact on women’s fertility (108) and no major obstetric or fetal complications emerged in exposed pregnancies (109–111). Patients switching to natalizumab for ART or already on treatment with this drug should continue the therapy during ART and pregnancy in order to minimize the risk of unwarranted relapses due to treatment suspension. The same approach should be proposed to women with MS treated with injectables compatible with pregnancy, given that IFNs or glatiramer acetate are not expected to have a detrimental impact on fertility or ART outcomes. Conversely, Bove et al. have shown that no treatment or treatment washout >3 months before ART increases the risk of relapses (88). So, in case of women treated with first-line orals not compatible with pregnancy, switching to injectables before starting ART procedures seems like a safer approach compared to treatment interruption.

These statements received 25, 0, 21.74, and 8.7% disagreement or doubt (neither agree nor disagree), respectively. The motivations supporting disagreements are outlined in the Supplementary material. One expert affirmed that it would be difficult to justify a therapeutical switch for a reason not intrinsically linked to the disease; in addition, many of them reinforced the importance of evaluating the disease course/activity.

#### 3.2.5. Topic 5. Oocyte cryopreservation in women affected by MS

S22. Oocyte cryopreservation should be considered in women with reduced ovarian reserve, who require unpredictable time to complete diagnosis workup and achieve control of the disease.

S23. Oocyte cryopreservation should be proposed to women with MS that must postpone pregnancy due to poor disease control that requires highly effective treatments not compatible with conception.

S24. In women above 35 years of age, the option of oocyte cryopreservation should be evaluated in the light of the ovarian reserve of the women and the risk-cost/benefit analysis.

The statements concerning oocyte cryopreservation reached 82.61, 91.31, and 68.19% agreement, respectively. The first to suggest fertility preservation in MS patients was Cavalla et al. (26) in 2006. They proposed that, similarly to cancer patients, people with MS could take advantage of this technology to preserve their fertility potential before starting treatments. Recently, Massarotti et al. (96) reinforced this suggestion, stressing the concept that, in light of the many unanswered questions about fertility and assisted reproduction outcomes in patients with multiple sclerosis, multidisciplinary counseling and dedicated clinics should be put in place to manage these aspects over time. Oocyte or embryo cryopreservation is already a consolidated practice for fertile women diagnosed with cancer before starting anti-neoplastic treatments such as chemotherapy and/or radiotherapy (112, 113). This is because such treatments are gonadotoxic, and therefore, the ovarian reserve and follicular pool could be dramatically reduced after certain oncologic protocols (114). Likewise, some MS treatments could be cytotoxic (i.e., mitoxantrone and cyclophosphamide), with effects also on gonads, although the impact of MS drugs on fertility is still a matter of debate for the majority of them (45, 115). For these reasons, MS could be considered a condition in which the preservation of gametes before therapy or during diagnostic workup could be considered, mainly in women showing signs of reduced fertility or in those patients who are candidates for gonadotoxic therapies such as bone marrow or stem cell transplantation. Data on fertility after autologous hematopoietic stem cell transplantation (aHSCT), which usually requires a conditioning regimen with alkylating agents, are still inconclusive. A study by Massarotti et al. (116) has shown that 70% of women recovered menses after treatment, especially if they were young, while Zafeiri et al. (117) observed a significant reduction of the AMH levels after the procedure. However, cryopreservation of ovarian tissue in eight women undergoing aHSCT resulted in good recovery of ovarian function in two women out of four with premature ovarian insufficiency after treatment (118). Despite the fact that ART treatments have been associated with an increased risk of post-procedure relapse, the evidence of reduced risk, whenever therapy is not discontinued (as the Boston cohort showed in the study from Bove et al. (88)), could open a new window in fertility preservation for women affected by MS, even if pregnancy is not advisable due to poor disease control. Gulekli et al. (119) were the first to report two cases of infertile women affected by MS who were treated with *in vitro* maturation (IVM) of oocytes to avoid the risk of ovarian stimulation and the related risk of disease reactivation, with successful pregnancy and live birth. The authors, therefore, suggested that MS would be considered an indication for this ART strategy. IVM could be a useful strategy for fertility preservation, in the centers that are familiar with this strategy, eventually in cases when disease control has not been achieved yet or when patients require highly effective treatments that are not compatible with conception. Finally, it is obvious that a risk/benefit analysis should be carried out by a multidisciplinary team in MS women of advanced reproductive age (e.g., over 35 years old), considering the markers of ovarian reserve. These statements received 17.39, 8.69, and 31.81% disagreement or doubt (neither agree nor disagree), respectively. The motivations supporting these disagreements are outlined in the Supplementary material. The main arguments commented on were that a long period for MS diagnosis is not acceptable or usually requested and that the decision on oocyte cryopreservation should always depend on the patient’s age.

## 4. Discussion

This Delphi consensus, with 21 statements approved, provides a real-world clinical perspective on the specific approaches during key steps of fertility assessment and ART management from a diverse group of Italian experts. What emerges is that the evaluation of female and couple fertility is gaining more and more importance in light of the possibility of supporting pregnancy desire and motherhood with therapeutic regimens that are proving to be effective and safe, although further evidence is still urgently needed. The Delphi methodology represents the strength of this study, which also benefited from the knowledge of an Italian panel of highly respected experts in the field. The fertility experts participating in the consensus were from a diverse range of global regions of Italy, including different fertility centers from the northern, middle, and southern Italy, reflecting the quality of healthcare and different approaches to infertility treatment. The consensus allowed to include of a wider list of topics than what would be typically considered in a systematic review or in a guideline, which are usually based on strict methodology, limiting the scope of the investigation. However, there are a few limitations; first, the consensus does not represent an exhaustive list of statements, and the statements only represent the collective opinion of the experts included. The majority of the statements reached consensus (more than 66% agreement) at first voting, with only 3 statements rejected out of 24. Some statements reached consensus even though a few experts disagreed with them (motivations in Supplementary materials), while one was approved unanimously. Moreover, the statements have been conceived taking into account the evidence from the literature, which is quite heterogeneous and limited by the small sample size of the studies performed so far, especially in regard to ovarian reserve estimation and assisted reproduction outcomes in women with MS. At last, although these statements represent the point of view of the experts, individualized management with regards to treatment options should always be planned in relation to patients needs and clinical features. Dobson et al., in 2019, released the guidelines of the Association of British Neurologists regarding MS and pregnancy, developed through a Delphi consensus. They focused on the evidence about all the DMDs in relation to contraception, fertility, pregnancy, and lactation, but mainly on general management of pre-, during, and post-pregnancy times for MS women. Importantly, they stated that pre-pregnancy counseling should be organized at diagnosis or soon after it, and eventually repeated yearly in women of reproductive age. Moreover, they admitted that ART procedures are not contraindicated, although a multidisciplinary team should plan how to effectively manage the patient during those times (120). A recent consensus came also from Argentina, with 50 statements: they were of the same advice regarding the need for reproductive counseling before pregnancy and at regular (annual) intervals, as well as the possibility of asking for ART procedures to get pregnant. An interesting recommendation they released is to seek a fertility specialist if conception does not happen after 6 months of attempts when DMDs have been stopped, instead of the classical 12 months usually considered for infertility in the general population (121). Another consensus has been provided by the Portuguese Multiple Sclerosis Study Group, which has issued a list of statements similar to the others previously mentioned, analyzing the needs of MS women from the pre-conceptional period to the post-partum period. Similarly, they addressed the fertility issue, admitting that the evidence does not support a role for both the disease and related treatments in the determination of infertility. Furthermore, they confirmed the possibility of seeking ART treatments to get pregnant (122). Very recently, Oreja-Guevara et al. published some recommendations for ART in MS by a Spanish expert panel (123). The main arguments were the need to assess the partner’s health and subfertility factors other than age, to consider less than 1 year of regular intercourse for infertility consultation after 35 years of age, single embryo transfer, and a maximum of three cycles of ovarian stimulation, and that a fertility preservation is a possible strategy. However, our consensus preferred to highlight the importance of proper fertility assessment and counseling, especially in relation to female age and the intrinsic reduction of fertility with aging, which prompts evaluation of fertility before it is too late. Although the reports on ovarian reserve suggested that highly active disease could cause impairment of AMH levels or AFC (12, 33), we suggest considering couple fertility evaluation to help speed up the family planning process, independently from disease activity, so as to reduce the time to pregnancy. Nonetheless, we suggested the use of oocyte cryopreservation in selected cases. Further evidence is still urgently needed on the issue of fertility and ART treatments in women affected by MS, since the number of them with reproductive desire is increasing but the mean age at family planning request could be close to a window of reduced fertility, and therefore both neurologists involved in MS care and reproductive medicine experts should manage these aspects over time.

## 5. Conclusion

This Delphi consensus provides 21 statements by expert opinions on specific approaches during the neurological assessment of women diagnosed with multiple sclerosis, including fertility evaluation, assisted reproduction, and fertility preservation, especially when women are older than 35 years old, with the aim of reducing the time to pregnancy.

## Data availability statement

The raw data supporting the conclusions of this article will be made available by the authors, without undue reservation.

## Ethics statement

Ethical review and approval was not required for the study on human participants in accordance with the local legislation and institutional requirements. Written informed consent from the participants or participants’ legal guardian/next of kin was not required to participate in this study in accordance with the national legislation and the institutional requirements.

## Group member of Interdisciplinary Group for Fertility in Multiple Sclerosis

Carlo Alviggi and Raffaella Di Girolamo, Department of Public Health, School of Medicine, University of Naples Federico II, Naples, Italy; Luigi Carbone and Roberta Lanzillo, Department of Neuroscience, Reproductive Sciences and Dentistry, School of Medicine, University of Naples Federico II, Naples, Italy; Doriana Landi and Girolama Alessandra Marfia, Multiple Sclerosis Clinical and Research Unit, University Hospital of Rome Tor Vergata, Rome, Italy; Paola Anserini, UOS Physiopathology of Human Reproduction, IRCCS Ospedale Policlinico San Martino, Genova, Italy; Diego Centonze, Department of Systems Medicine, Laboratory of Synaptic Immunopathology, “Tor Vergata” University, Rome, Italy; Unit of Neurology–IRCCS Neuromed, Pozzilli, Isernia, Italy; Pietro Annovazzi, Multiple Sclerosis Center, Hospital of Gallarate–ASST della Valle Olona, Gallarate, Italy; Simona Bonavita, Department of Advanced Medical and Surgical Sciences, University of Campania Luigi Vanvitelli, Naples, Italy; Giovanna Borriello, Multiple Sclerosis Center, ‘S. Andrea’ Hospital, Sapienza University of Rome, Rome, Italy; Paola Cavalla, Multiple Sclerosis Center, City of Health and Science University Hospital, Turin, Italy; Raffaella Cerqua, Neurological Clinic, Department of Experimental and Clinical Medicine, Ospedali Riuniti, Ancona, Italy; Marinella Clerico, Clinical and Biological Sciences Department, Neurology Unit, University of Torino, ‘San Luigi Gonzaga’ Hospital, Orbassano, Italy; Eleonora Cocco, Multiple Sclerosis Center, Department of Medical Science and Public Health, University of Cagliari, Cagliari, Italy; Cinzia Cordioli, Multiple Sclerosis Center, Spedali Civili of Brescia, Montichiari (BS), Italy; Emanuele D’Amico, Department of Medical and Surgical Sciences and Advanced Technologies ‘G.F. Ingrassia’, Section of Neurosciences, University of Catania, Catania, Italy; Giovanna De Luca, Multiple Sclerosis Center, ‘SS Annunziata’ Hospital, ‘Gabriele d’Annunzio’ University Chieti-Pescara, Chieti, Italy; Massimiliano Di Filippo, Section of Neurology, Department of Medicine and Surgery, University of Perugia, Perugia, Italy; Roberta Fantozzi, Unit of Neurology–IRCCS Neuromed, Pozzilli, Isernia, Italy; Diana Ferraro, Department of Neuroscience, UO of Neurology, AOU Policlinico OB, Modena, Italy; Pietro Iaffaldano, Department of Basic Medical Sciences, Neurosciences and Sense Organs, University of Bari Aldo Moro, Bari/Italy; Matilde Inglese, Department of Neuroscience, Rehabilitation, Ophthalmology, Genetics, Maternal and Child Health (DiNOGMI), University of Genoa, Genova, Italy; Department of Neurology, Policlinico ‘San Martino Hospital’-Sistema Sanitario Regione, Genoa, Italy; Paola Perini, Multiple Sclerosis Center, Neurological Clinic, University Hospital of Padua, Italy; Emilio Portaccio, Division Neurological Rehabilitation, Department of NEUROFARBA, University of Florence, Florence, Italy; Paolo Ragonese, Unit of Neurology, Department of Biomedicine, Neurosciences and Advanced Diagnostics, Palermo University, Palermo, Italy; Cinzia Scandellari, IRCCS Institute of Neurological Sciences, UOSI Multiple Sclerosis Rehabilitation, Bologna, Italy; Valentina Torri Clerici, Neuroimmunology and Neuromuscular Diseases Unit, Fondazione I.R.C.C.S. Istituto Neurologico Carlo Besta, Milan, Italy; Carla Tortorella, Department of Neurosciences, S. Camillo-Forlanini Hospital, Rome, Italy; Paola Valentino, Department of Medical and Surgical Sciences, Institute of Neurology, Magna Graecia University, Catanzaro, Italy.

## Author contributions

LC: Visualization, Writing – review and editing. DL: Visualization, Writing – review and editing. RDG: Data curation, Writing – original draft. PA: Validation, Writing – review and editing. DC: Validation, Writing – review and editing. GM: Conceptualization, Supervision, Writing – review and editing. CA: Conceptualization, Methodology, Supervision, Visualization, Writing – review and editing.

## References

[1] Ysrraelit MC Correale J. Impact of sex hormones on immune function and multiple sclerosis development. Immunology. (2019) 156:9–22. doi: 10.1111/imm.13004, PMID: 30222193 PMC6283654

[2] Ghezzi A Zaffaroni M. Female-specific issues in multiple sclerosis. Expert Rev Neurother. (2008) 8:969–77. doi: 10.1586/14737175.8.6.969, PMID: 18505361

[3] Wang G Marrie RA Fox RJ Tyry T Cofield SS Cutter GR. Treatment satisfaction and bothersome bladder, bowel, sexual symptoms in multiple sclerosis. Mult Scler Relat Disord. (2018) 20:16–21. doi: 10.1016/j.msard.2017.12.006, PMID: 29275057

[4] Nguyen AL Havrdova EK Horakova D Izquierdo G Kalincik T Van der Walt A. Incidence of pregnancy and disease-modifying therapy exposure trends in women with multiple sclerosis: a contemporary cohort study. Mult Scler Relat Disord. (2019) 28:235–43. doi: 10.1016/j.msard.2019.01.003, PMID: 30623864

[5] Confavreux C Hutchinson M Hours MM Cortinovis-Tourniaire P Moreau T. Rate of pregnancy-related relapse in multiple sclerosis. Pregnancy in multiple sclerosis group. N Engl J Med. (1998) 339:285–91. doi: 10.1056/NEJM199807303390501, PMID: 9682040

[6] Krysko KM Graves JS Dobson R Altintas A Amato MP Bernard J. Sex effects across the lifespan in women with multiple sclerosis. Ther Adv Neurol Disord. (2020) 13:3616. doi: 10.1177/1756286420936166, PMID: 32655689 PMC7331774

[7] Roux T Courtillot C Debs R Touraine P Lubetzki C Papeix C. Fecundity in women with multiple sclerosis: an observational mono-centric study. J Neurol. (2015) 262:957–60. doi: 10.1007/s00415-015-7663-1, PMID: 25673128

[8] Houtchens M Kaplan T. Reproductive issues in MS. Semin Neurol. (2017) 37:632–42. doi: 10.1055/s-0037-1608925, PMID: 29270936

[9] Bonavita S Lavorgna L Worton H Russell S Jack D. Family planning decision making in people with multiple sclerosis. Front Neurol. (2021) 12:620772. doi: 10.3389/fneur.2021.620772, PMID: 33995240 PMC8113643

[10] Thöne J Kleiter I Stahl A Ellrichmann G Gold R Hellwig K. Relevance of endoglin, IL-1α, IL-1β and anti-ovarian antibodies in females with multiple sclerosis. J Neurol Sci. (2016) 362:240–3. doi: 10.1016/j.jns.2016.01.057, PMID: 26944156

[11] Thöne J Kollar S Nousome D Ellrichmann G Kleiter I Gold R. Serum anti-Müllerian hormone levels in reproductive-age women with relapsing–remitting multiple sclerosis. Mult Scler. (2015) 21:41–7. doi: 10.1177/1352458514540843, PMID: 25145691

[12] Sepúlveda M Ros C Martínez-Lapiscina EH Solà-Valls N Hervàs M Llufriu S. Pituitary-ovary axis and ovarian reserve in fertile women with multiple sclerosis: a pilot study. Mult Scler. (2016) 22:564–8. doi: 10.1177/1352458515602339, PMID: 26362892

[13] Sadeghpour N Mirmosayyeb O Bjørklund G Shaygannejad V. Is fertility affected in women of childbearing age with multiple sclerosis or Neuromyelitis Optica Spectrum disorder? J Mol Neurosci. (2020) 70:1829–35. doi: 10.1007/s12031-020-01576-x, PMID: 32740781

[14] Cil AP Leventoğlu A Sönmezer M Soylukoç R Oktay K. Assessment of ovarian reserve and Doppler characteristics in patients with multiple sclerosis using immunomodulating drugs. J Turk Ger Gynecol Assoc. (2009) 10:213–9. PMID: 24591875 PMC3939168

[15] Kirshenbaum M Orvieto R. Premature ovarian insufficiency (POI) and autoimmunity-an update appraisal. J Assist Reprod Genet. (2019) 36:2207–15. doi: 10.1007/s10815-019-01572-0, PMID: 31440958 PMC6885484

[16] Jølving LR Larsen MD Fedder J Nørgård BM. Live birth in women with multiple sclerosis receiving assisted reproduction. Reprod Biomed Online. (2020) 40:711–8. doi: 10.1016/j.rbmo.2020.01.013, PMID: 32317230

[17] Eisenberg ML Thoma ME Li S McLain AC. Trends in time-to-pregnancy in the USA: 2002 to 2017. Hum Reprod. (2021) 36:2331–8. doi: 10.1093/humrep/deab107, PMID: 34021350 PMC8289323

[18] Smarr MM Sapra KJ Gemmill A Kahn LG Wise LA Lynch CD. Is human fecundity changing? A discussion of research and data gaps precluding us from having an answer. Hum Reprod. (2017) 32:499–504. doi: 10.1093/humrep/dew361, PMID: 28137753 PMC5850610

[19] American College of Obstetricians and Gynecologists Committee on Gynecologic Practice and Practice Committee. Female age-related fertility decline. Committee opinion no. 589. Fertil Steril. (2014) 101:633–4. doi: 10.1016/j.fertnstert.2013.12.032, PMID: 24559617

[20] Alviggi C Humaidan P Howles CM Tredway D Hillier SG. Biological versus chronological ovarian age: implications for assisted reproductive technology. Reprod Biol Endocrinol. (2009) 7:101. doi: 10.1186/1477-7827-7-101, PMID: 19772632 PMC2764709

[21] Esteves SC Alviggi C Humaidan P Fischer R Andersen CY Conforti A. The POSEIDON criteria and its measure of success through the eyes of clinicians and embryologists. Front Endocrinol (Lausanne). (2019) 10:814. doi: 10.3389/fendo.2019.00814, PMID: 31824427 PMC6880663

[22] Haahr T Dosouto C Alviggi C Esteves SC Humaidan P. Management strategies for POSEIDON groups 3 and 4. Front Endocrinol (Lausanne). (2019) 10:614. doi: 10.3389/fendo.2019.00614, PMID: 31572298 PMC6749147

[23] Korsholm AS Petersen KB Bentzen JG Hilsted LM Andersen AN Hvidman HW. Investigation of anti-Müllerian hormone concentrations in relation to natural conception rate and time to pregnancy. Reprod Biomed Online. (2018) 36:568–75. doi: 10.1016/j.rbmo.2018.01.013, PMID: 29478840

[24] Frick AP. Advanced maternal age and adverse pregnancy outcomes. Best Pract Res Clin Obstet Gynaecol. (2021) 70:92–100. doi: 10.1016/j.bpobgyn.2020.07.005, PMID: 32741623

[25] Di Girolamo R D’Antonio F. Infertility and adverse pregnancy outcome: from pathophysiology to clinical relevance. Minerva Obstet Gynecol. (2021) 74:1–2. doi: 10.23736/S2724-606X.21.05021-1, PMID: 34904589

[26] Cavalla P Rovei V Masera S Vercellino M Massobrio M Mutani R. Fertility in patients with multiple sclerosis: current knowledge and future perspectives. Neurol Sci. (2006) 27:231–9. doi: 10.1007/s10072-006-0676-x, PMID: 16998725

[27] Houtchens MK Edwards NC Hayward B Mahony MC Phillips AL. Live birth rates, infertility diagnosis, and infertility treatment in women with and without multiple sclerosis: data from an administrative claims database. Mult Scler Relat Disord. (2020) 46:102541. doi: 10.1016/j.msard.2020.102541, PMID: 33296964

[28] Jalkanen A Alanen A Airas L. Finnish multiple sclerosis and pregnancy study group. Pregnancy outcome in women with multiple sclerosis: results from a prospective nationwide study in Finland. Mult Scler. (2010) 16:950–5. doi: 10.1177/1352458510372629, PMID: 20542921

[29] McCombe PA Stenager E. Female infertility and multiple sclerosis: is this an issue? Mult Scler. (2015) 21:5–7. doi: 10.1177/1352458514549406 25583835

[30] Finkelsztejn A Brooks JB Paschoal FM Fragoso YD. What can we really tell women with multiple sclerosis regarding pregnancy? A systematic review and meta-analysis of the literature. BJOG. (2011) 118:790–7. doi: 10.1111/j.1471-0528.2011.02931.x, PMID: 21401856

[31] Lavorgna L Esposito S Lanzillo R Sparaco M Ippolito D Cocco E. Factors interfering with parenthood decision-making in an Italian sample of people with multiple sclerosis: an exploratory online survey. J Neurol. (2019) 266:707–16. doi: 10.1007/s00415-019-09193-4, PMID: 30649617

[32] Ferraro D Simone AM Adani G Vitetta F Mauri C Strumia S. Definitive childlessness in women with multiple sclerosis: a multicenter study. Neurol Sci. (2017) 38:1453–9. doi: 10.1007/s10072-017-2999-1, PMID: 28536948

[33] Graves JS Henry RG Cree BAC Lambert-Messerlian G Greenblatt RM Waubant E. Ovarian aging is associated with gray matter volume and disability in women with MS. Neurology. (2018) 90:e254–60. doi: 10.1212/WNL.0000000000004843, PMID: 29273686 PMC5772165

[34] Carbone L Di Girolamo R Conforti A Iorio GG Simeon V Landi D. Ovarian reserve in patients with multiple sclerosis: a systematic review and meta-analysis. Int J Gynaecol Obstet. (2023) 2023:14757. doi: 10.1002/ijgo.14757, PMID: 37017322

[35] Katz SI. Classification, diagnosis, and differential diagnosis of multiple sclerosis. Curr Opin Neurol. (2015) 28:193–205. doi: 10.1097/WCO.0000000000000206, PMID: 25887774

[36] Mathews TJ Hamilton BE. Mean age of mothers is on the rise: United States, 2000-2014. NCHS Data Brief. (2016) 232:1–8. PMID: 26828319

[37] Wundes A Pebdani RN Amtmann D. What do healthcare providers advise women with multiple sclerosis regarding pregnancy? Mult Scler Int. (2014) 2014:819216. doi: 10.1155/2014/819216, PMID: 24872893 PMC3964846

[38] Sadovnick D Criscuoli M Yee I Carruthers R Schabas A Smyth P. The road to conception for women with multiple sclerosis. Mult Scler J Exp Transl Clin. (2021) 7:2313. doi: 10.1177/20552173211032313, PMID: 34350028 PMC8287372

[39] Bove R Alwan S Friedman JM Hellwig K Houtchens M Koren G. Management of multiple sclerosis during pregnancy and the reproductive years: a systematic review. Obstet Gynecol. (2014) 124:1157–68. doi: 10.1097/AOG.0000000000000541, PMID: 25415167

[40] Vukusic S Marignier R. Multiple sclerosis and pregnancy in the 'treatment era'. Nat Rev Neurol. (2015) 11:280–9. doi: 10.1038/nrneurol.2015.53, PMID: 25896084

[41] Coyle PK Oh J Magyari M Oreja-Guevara C Houtchens M. Management strategies for female patients of reproductive potential with multiple sclerosis: an evidence-based review. Mult Scler Relat Disord. (2019) 32:54–63. doi: 10.1016/j.msard.2019.04.003, PMID: 31030020

[42] Canibaño B Deleu D Mesraoua B Melikyan G Ibrahim F Hanssens Y. Pregnancy-related issues in women with multiple sclerosis: an evidence-based review with practical recommendations. J Drug Assess. (2020) 9:20–36. doi: 10.1080/21556660.2020.1721507, PMID: 32128285 PMC7034025

[43] Fragoso YD Adoni T Brooks JBB Finkelsztejn A da Gama PD Grzesiuk AK. Practical evidence-based recommendations for patients with multiple sclerosis who want to have children. Neurol Ther. (2018) 7:207–32. doi: 10.1007/s40120-018-0110-3, PMID: 30167914 PMC6283793

[44] Krysko KM Bove R Dobson R Jokubaitis V Hellwig K. Treatment of women with multiple sclerosis planning pregnancy. Curr Treat Options Neurol. (2021) 23:11. doi: 10.1007/s11940-021-00666-4, PMID: 33814892 PMC8008016

[45] Amato MP Portaccio E. Fertility, pregnancy and childbirth in patients with multiple sclerosis: impact of disease-modifying drugs. CNS Drugs. (2015) 29:207–20. doi: 10.1007/s40263-015-0238-y, PMID: 25773609

[46] World Health Organization. International classification of diseases, 11th revision (ICD-11). Geneva: World Health Organization (2018).

[47] Alroughani R Alowayesh MS Ahmed SF Behbehani R Al-Hashel J. Relapse occurrence in women with multiple sclerosis during pregnancy in the new treatment era. Neurology. (2018) 90:e840–6. doi: 10.1212/WNL.0000000000005065, PMID: 29429970

[48] Hughes SE Spelman T Gray OM Boz C Trojano M Lugaresi A. Predictors and dynamics of postpartum relapses in women with multiple sclerosis. Mult Scler. (2014) 20:739–46. doi: 10.1177/1352458513507816, PMID: 24107309

[49] Bsteh G Algrang L Hegen H Auer M Wurth S di Pauli F. Pregnancy and multiple sclerosis in the DMT era: a cohort study in Western Austria. Mult Scler. (2020) 26:69–78. doi: 10.1177/1352458518816614, PMID: 30507345

[50] Montalban X Gold R Thompson AJ Otero-Romero S Amato MP Chandraratna D. ECTRIMS/EAN guideline on the pharmacological treatment of people with multiple sclerosis. Mult Scler. (2018) 24:96–120. doi: 10.1177/1352458517751049, PMID: 29353550

[51] European Medicines Agency. *Rebif - summary of product characteristics*. (2022). Available at: https://www.ema.europa.eu/en/documents/product-information/rebif-epar-product-information_en.pdf (Accessed September, 25, 2022).

[52] European Medicines Agency. *Plegridy - summary of product characteristics*. (2022). Available at: https://www.ema.europa.eu/en/documents/product-information/plegridy-epar-product-information_en.pdf (Accessed September 25, 2022).

[53] European Medicines Agency. *Betaferon - summary of product characteristics*. (2022). Available at: https://www.ema.europa.eu/en/documents/product-information/betaferon-epar-product-information_en.pdf (Accessed September 25, 2022).

[54] European Medicines Agency. *Extavia summary of product characteristics*. (2022). Available at: https://www.ema.europa.eu/en/documents/product-information/extavia-epar-product-information_it.pdf (Accessed September 25, 2022).

[55] European Medicines Agency. *Avonex - summary of product characteristics*. Available at: https://www.ema.europa.eu/en/documents/product-information/avonex-epar-product-information_it.pdf (Accessed September 25, 2022).

[56] Highlights of Prescribing Information: Copaxone. Available at: https://www.copaxone.com/globalassets/copaxone/prescribing-information.pdf (Accessed September 25, 2022).

[57] Amato MP Portaccio E Ghezzi A Hakiki B Zipoli V Martinelli V. Pregnancy and fetal outcomes after interferon-β exposure in multiple sclerosis. Neurology. (2010) 75:1794–802. doi: 10.1212/WNL.0b013e3181fd62bb, PMID: 21079181

[58] Patti F Cavallaro T Fermo S Nicoletti A Cimino V Vecchio R. Is in utero early-exposure to interferon beta a risk factor for pregnancy outcomes in multiple sclerosis? J Neurol. (2008) 255:1250–3. doi: 10.1007/s00415-008-0909-4, PMID: 18677640

[59] Thiel S Langer-Gould A Rockhoff M Haghikia A Queisser-Wahrendorf A Gold R. Interferon-beta exposure during first trimester is safe in women with multiple sclerosis-a prospective cohort study from the German multiple sclerosis and pregnancy registry. Mult Scler. (2016) 22:801–9. doi: 10.1177/1352458516634872, PMID: 26920382

[60] Hellwig K Duarte Caron F Wicklein EM Bhatti A Adamo A. Pregnancy outcomes from the global pharmacovigilance database on interferon beta-1b exposure. Ther Adv Neurol Disord. (2020) 13:1756286420910310. doi: 10.1177/1756286420910310 32201504 PMC7066586

[61] Burkill S Vattulainen P Geissbuehler Y Sabido Espin M Popescu C Suzart-Woischnik K. The association between exposure to interferon-beta during pregnancy and birth measurements in offspring of women with multiple sclerosis. PLoS One. (2019) 14:e0227120. doi: 10.1371/journal.pone.0227120, PMID: 31887199 PMC6936848

[62] Giannini M Portaccio E Ghezzi A Hakiki B Pastò L Razzolini L. Pregnancy and fetal outcomes after Glatiramer acetate exposure in patients with multiple sclerosis: a prospective observational multicentric study. BMC Neurol. (2012) 12:124. doi: 10.1186/1471-2377-12-124, PMID: 23088447 PMC3487812

[63] Herbstritt S Langer-Gould A Rockhoff M Haghikia A Queisser-Wahrendorf A Gold R. Glatiramer acetate during early pregnancy: a prospective cohort study. Mult Scler. (2016) 22:810–6. doi: 10.1177/1352458515623366, PMID: 26754804

[64] Fragoso YD Finkelsztejn A Kaimen-Maciel DR Grzesiuk AK Gallina AS Lopes J. Long-term use of glatiramer acetate by 11 pregnant women with multiple sclerosis: a retrospective, multicentre case series. CNS Drugs. (2010) 24:969–76. doi: 10.2165/11538960-000000000-00000, PMID: 20806993

[65] Sabidó Espin M Suzart-Woischnik K Grimes N Prach LM Zhao L Hakkarainen KM. Inform - interferon Beta exposure in the 2nd and 3rd trimester of pregnancy: a register-based drug utilisation study in Finland and Sweden. Mult Scler Relat Disord. (2022) 59:103572. doi: 10.1016/j.msard.2022.103572

[66] Almouzain L Stevenson F Chard D Rahman NA Hamilton F. Switching treatments in clinically stable relapsing remitting multiple sclerosis patients planning for pregnancy. Mult Scler J Exp Transl Clin. (2021) 7:1571. doi: 10.1177/20552173211001571, PMID: 33796332 PMC7985951

[67] Villaverde-González R Candeliere-Merlicco A Alonso-Frías MA Aparicio Castro E Carrillo Alcaraz A Mallada Frechín J. Discontinuation of disease-modifying treatments in multiple sclerosis to plan a pregnancy: a retrospective registry study. Mult Scler Relat Disord. (2020) 46:102518. doi: 10.1016/j.msard.2020.102518, PMID: 32977075

[68] Portaccio E Moiola L Martinelli V Annovazzi P Ghezzi A Zaffaroni M. Pregnancy decision-making in women with multiple sclerosis treated with natalizumab. Neurology. (2018) 90:e832–9. doi: 10.1212/WNL.0000000000005068, PMID: 29438041

[69] Hellwig K Tokic M Thiel S Esters N Spicher C Timmesfeld N. Multiple sclerosis disease activity and disability following discontinuation of Natalizumab for pregnancy. JAMA Netw Open. (2022) 5:e2144750. doi: 10.1001/jamanetworkopen.2021.44750, PMID: 35072719 PMC8787598

[70] Bianco A Lucchini M Totaro R Fantozzi R de Luca G di Lemme S. Disease reactivation after Fingolimod discontinuation in pregnant multiple sclerosis patients. Neurotherapeutics. (2021) 18:2598–607. doi: 10.1007/s13311-021-01106-6, PMID: 34494237 PMC8803993

[71] Novi G Ghezzi A Pizzorno M Lapucci C Bandini F Annovazzi P. Dramatic rebounds of MS during pregnancy following fingolimod withdrawal. Neurol Neuroimmunol Neuroinflamm. (2017) 4:e377. doi: 10.1212/NXI.0000000000000377, PMID: 28804745 PMC5532747

[72] Lamaita R Melo C Laranjeira C Barquero P Gomes J Silva-Filho A. Multiple sclerosis in pregnancy and its role in female fertility: a systematic review. JBRA Assist Reprod. (2021) 25:493–9. doi: 10.5935/1518-0557.20210022 34061482 PMC8312296

[73] Krysko KM Dobson R Alroughani R Amato MP Bove R Ciplea AI. Family planning considerations in people with multiple sclerosis. Lancet Neurol. (2023) 22:350–66. doi: 10.1016/S1474-4422(22)00426-4 36931808

[74] Landi D Bovis F Grimaldi A Annovazzi PO Bertolotto A Bianchi A. Exposure to natalizumab throughout pregnancy: effectiveness and safety in an Italian cohort of women with multiple sclerosis. J Neurol Neurosurg Psychiatry. (2022) 2022:329657. doi: 10.1136/jnnp-2022-329657, PMID: 36180219

[75] Roos I Leray E Frascoli F Casey R Brown JWL Horakova D. Delay from treatment start to full effect of immunotherapies for multiple sclerosis. Brain. (2020) 143:2742–56. doi: 10.1093/brain/awaa231, PMID: 32947619

[76] Houtchens MK Edwards NC Schneider G Stern K Phillips AL. Pregnancy rates and outcomes in women with and without MS in the United States. Neurology. (2018) 91:e1559–69. doi: 10.1212/WNL.0000000000006384 30266889 PMC6205683

[77] European Medicines Agency. *Aubagio - summary of product characteristics*. Available at: https://www.ema.europa.eu/en/documents/product-information/aubagio-epar-product-information_en.pdf (Accessed September 25, 2022).

[78] European Medicines Agency. Mavenclad - summary of product characteristics [internet], https://www.ema.europa.eu/en/documents/product-information/mavenclad-epar-product-information_en.pdf (Accessed September 25, 2022).

[79] Baker D Pryce G Herrod SS Schmierer K. Potential mechanisms of action related to the efficacy and safety of cladribine. Mult Scler Relat Disord. (2019) 30:176–86. doi: 10.1016/j.msard.2019.02.018, PMID: 30785074

[80] Hellwig K Beste C Brune N Haghikia A Müller T Schimrigk S. Increased MS relapse rate during assisted reproduction technique. J Neurol. (2008) 255:592–3. doi: 10.1007/s00415-008-0607-2, PMID: 18408881

[81] Hellwig K Schimrigk S Beste C Müller T Gold R. Increase in relapse rate during assisted reproduction technique in patients with multiple sclerosis. Eur Neurol. (2009) 61:65–8. doi: 10.1159/000177937, PMID: 19039223

[82] Laplaud DA Leray E Barrière P Wiertlewski S Moreau T. Increase in multiple sclerosis relapse rate following in vitro fertilization. Neurology. (2006) 66:1280–1. doi: 10.1212/01.wnl.0000208521.10685.a6, PMID: 16636258

[83] Laplaud DA Lefrère F Leray E Barrière P Wiertlewski S. Augmentation du risque de poussée de sclérose en plaques après stimulation ovarienne pour fécondation in vitro [Increased risk of relapse in multiple sclerosis patients after ovarian stimulation for in vitro fertilization]. Gynecol Obstet Fertil. (2007) 35:1047–50. doi: 10.1016/j.gyobfe.2007.07.033, PMID: 17916439

[84] Michel L Foucher Y Vukusic S Confavreux C de Sèze J Brassat D. Club francophone de la Sclérose En plaques (CFSEP). Increased risk of multiple sclerosis relapse after in vitro fertilisation. J Neurol Neurosurg Psychiatry. (2012) 83:796–802. doi: 10.1136/jnnp-2012-302235, PMID: 22693287

[85] Correale J Farez MF Ysrraelit MC. Increase in multiple sclerosis activity after assisted reproduction technology. Ann Neurol. (2012) 72:682–94. doi: 10.1002/ana.23745, PMID: 23034952

[86] Ladwig A Dunkl V Richter N Schroeter M. Two cases of multiple sclerosis manifesting after in vitro fertilization procedures. J Neurol. (2016) 263:603–5. doi: 10.1007/s00415-016-8041-3, PMID: 26886203

[87] Torkildsen Ø Holmøy T Myhr KM. Severe multiple sclerosis reactivation after gonadotropin treatment. Mult Scler Relat Disord. (2018) 22:38–40. doi: 10.1016/j.msard.2018.02.031, PMID: 29525298

[88] Bove R Rankin K Lin C Zhao C Correale J Hellwig K. Effect of assisted reproductive technology on multiple sclerosis relapses: case series and meta-analysis. Mult Scler. (2020) 26:1410–9. doi: 10.1177/1352458519865118, PMID: 31368394

[89] Shimizu Y Ikeguchi R Kitagawa K. Pregnancy outcome, changes in lymphocyte subsets in peripheral blood, and plasma osteopontin in Japanese patients with multiple sclerosis after assisted reproductive technology (P4.369). Neurology. (2018) 90:369.

[90] Mainguy M Tillaut H Degremont A le Page E Mainguy C Duros S. Assessing the risk of relapse requiring corticosteroids after in vitro fertilization in women with multiple sclerosis. Neurology. (2022) 99:e1916–25. doi: 10.1212/WNL.0000000000201027, PMID: 35953288

[91] Graham EL Bakkensen JB Anderson A Lancki N Davidson A Perez Giraldo G. Inflammatory activity after diverse fertility treatments: a multicenter analysis in the modern multiple sclerosis treatment era. Neurol Neuroimmunol Neuroinflamm. (2023) 10:e200106. doi: 10.1212/NXI.0000000000200106, PMID: 36922025 PMC10018493

[92] Stanhiser J Steiner AZ. Psychosocial aspects of fertility and assisted reproductive technology. Obstet Gynecol Clin N Am. (2018) 45:563–74. doi: 10.1016/j.ogc.2018.04.006, PMID: 30092929

[93] Gameiro S Boivin J Dancet E de Klerk C Emery M Lewis-Jones C. ESHRE guideline: routine psychosocial care in infertility and medically assisted reproduction-a guide for fertility staff. Hum Reprod. (2015) 30:2476–85. doi: 10.1093/humrep/dev177, PMID: 26345684

[94] Verhaak CM Lintsen AM Evers AW Braat DD. Who is at risk of emotional problems and how do you know? Screening of women going for IVF treatment. Hum Reprod. (2010) 25:1234–40. doi: 10.1093/humrep/deq054, PMID: 20228392

[95] Van Der Walt A Nguyen AL Jokubaitis V. Family planning, antenatal and post partum care in multiple sclerosis: a review and update. Med J Aust. (2019) 211:230–6. doi: 10.5694/mja2.50113, PMID: 30919466

[96] Massarotti C Inglese M Anserini P. Fertility in multiple sclerosis patients: still many unanswered questions. Reprod Biomed Online. (2020) 41:567. doi: 10.1016/j.rbmo.2020.06.003, PMID: 32622704

[97] Esteves SC Roque M Bedoschi GM Conforti A Humaidan P Alviggi C. Defining low prognosis patients undergoing assisted reproductive technology: POSEIDON criteria-the why. Front Endocrinol (Lausanne). (2018) 9:461. doi: 10.3389/fendo.2018.00461, PMID: 30174650 PMC6107695

[98] Conforti A Esteves SC Cimadomo D Vaiarelli A di Rella F Ubaldi FM. Management of Women with an unexpected low ovarian response to gonadotropin. Front Endocrinol (Lausanne). (2019) 10:387. doi: 10.3389/fendo.2019.00387, PMID: 31316461 PMC6610322

[99] Esteves SC Conforti A Sunkara SK Carbone L Picarelli S Vaiarelli A. Improving reporting of clinical studies using the POSEIDON criteria: POSORT guidelines. Front Endocrinol (Lausanne). (2021) 12:587051. doi: 10.3389/fendo.2021.587051, PMID: 33815269 PMC8017440

[100] Esteves SC Yarali H Ubaldi FM Carvalho JF Bento FC Vaiarelli A. Validation of ART calculator for predicting the number of metaphase II oocytes required for obtaining at least one Euploid blastocyst for transfer in couples undergoing in vitro fertilization/intracytoplasmic sperm injection. Front Endocrinol (Lausanne). (2020) 10:917. doi: 10.3389/fendo.2019.00917, PMID: 32038484 PMC6992582

[101] Esteves SC Carvalho JF Martinhago CD Melo AA Bento FC Humaidan P. POSEIDON (patient-oriented strategies encompassing IndividualizeD oocyte number) group. Estimation of age-dependent decrease in blastocyst euploidy by next generation sequencing: development of a novel prediction model. Panminerva Med. (2019) 61:3–10. doi: 10.23736/S0031-0808.18.03507-3, PMID: 29962186

[102] Casella C Carbone L Conforti A Marrone V Cioffi G Buonfantino C. Preimplantation genetic testing: comparative analysis of jurisprudential regulations. Ital J Gynaecol Obstet. (2020) 32:237–47. doi: 10.36129/jog.32.04.03

[103] Vaiarelli A Cimadomo D Argento C Ubaldi N Trabucco E Drakopoulos P. Double stimulation in the same ovarian cycle (DuoStim) is an intriguing strategy to improve oocyte yield and the number of competent embryos in a short timeframe. Minerva Ginecol. (2019) 71:372–6. doi: 10.23736/S0026-4784.19.04390-9, PMID: 30848112

[104] Vaiarelli A Cimadomo D Gennarelli G Guido M Alviggi C Conforti A. Second stimulation in the same ovarian cycle: an option to fully-personalize the treatment in poor prognosis patients undergoing PGT-A. J Assist Reprod Genet. (2022) 39:663–73. doi: 10.1007/s10815-022-02409-z, PMID: 35128583 PMC8995212

[105] Esteves SC Roque M Sunkara SK Conforti A Ubaldi FM Humaidan P. Oocyte quantity, as well as oocyte quality, plays a significant role for the cumulative live birth rate of a POSEIDON criteria patient. Hum Reprod. (2019) 34:2555–7. doi: 10.1093/humrep/dez181, PMID: 31756248

[106] Vaiarelli A Cimadomo D Petriglia C Conforti A Alviggi C Ubaldi N. DuoStim - a reproducible strategy to obtain more oocytes and competent embryos in a short time-frame aimed at fertility preservation and IVF purposes. A systematic review. Ups J Med Sci. (2020) 125:121–30. doi: 10.1080/03009734.2020.1734694, PMID: 32338123 PMC7721001

[107] Landi D Ragonese P Prosperini L Nociti V Haggiag S Cortese A. Abortion induces reactivation of inflammation in relapsing-remitting multiple sclerosis. J Neurol Neurosurg Psychiatry. (2018) 89:1272–8. doi: 10.1136/jnnp-2018-318468, PMID: 29970387

[108] European Medicines Agency. *Tysabri - summary of product characteristics*. Available at: https://www.ema.europa.eu/en/documents/product-information/tysabri-epar-product-information_en.pdf (Accessed September, 25, 2022).

[109] Landi D Marfia GA. Exposure to natalizumab during pregnancy and lactation is safe - yes. Mult Scler. (2020) 26:887–9. doi: 10.1177/1352458520915814, PMID: 32508253

[110] Triplett JD Vijayan S Rajanayagam S Tuch P Kermode AG. Pregnancy outcomes amongst multiple sclerosis females with third trimester natalizumab use. Mult Scler Relat Disord. (2020) 40:101961. doi: 10.1016/j.msard.2020.101961, PMID: 32028118

[111] Peng A Qiu X Zhang L Zhu X He S Lai W. Natalizumab exposure during pregnancy in multiple sclerosis: a systematic review. J Neurol Sci. (2019) 396:202–5. doi: 10.1016/j.jns.2018.11.026, PMID: 30502611

[112] Harada M Osuga Y. Fertility preservation for female cancer patients. Int J Clin Oncol. (2019) 24:28–33. doi: 10.1007/s10147-018-1252-0, PMID: 29502284

[113] Cariati F Carbone L Iorio GG Conforti A Capasso A Bagnulo F. Cryopreservation of ovarian tissue: the biggest challenge of oncofertility. Minerva. Obstet Gynecol. (2022) 75:371–8. doi: 10.23736/S2724-606X.22.05066-7. Epub ahead of print, PMID: 35420290

[114] Spears N Lopes F Stefansdottir A Rossi V de Felici M Anderson RA. Ovarian damage from chemotherapy and current approaches to its protection. Hum Reprod Update. (2019) 25:673–93. doi: 10.1093/humupd/dmz027, PMID: 31600388 PMC6847836

[115] Cocco E Sardu C Gallo P Capra R Amato MP Trojano M. Frequency and risk factors of mitoxantrone-induced amenorrhea in multiple sclerosis: the FEMIMS study. Mult Scler. (2008) 14:1225–33. doi: 10.1177/1352458508094882, PMID: 18701568

[116] Massarotti C Sbragia E Boffa G Vercelli C Zimatore GB Cottone S. Menstrual cycle resumption and female fertility after autologous hematopoietic stem cell transplantation for multiple sclerosis. Mult Scler. (2021) 27:2103–7. doi: 10.1177/13524585211000616, PMID: 33709839

[117] Zafeiri L Åkerfeldt T Tolf A Carlson K Skalkidou A Burman J. Anti-Müllerian hormone and pregnancy after autologous hematopoietic stem cell transplantation for multiple sclerosis. PLoS One. (2023) 18:e0284288. doi: 10.1371/journal.pone.0284288, PMID: 37043510 PMC10096464

[118] Colmorn LB Kristensen SG Larsen EC Macklon KT. Cryopreservation of ovarian tissue as fertility preservation in young women with multiple sclerosis before stem cell transplantation. Mult Scler Relat Disord. (2023) 74:104716. doi: 10.1016/j.msard.2023.104716, PMID: 37087965

[119] Gulekli B Kovali Sezer M Gungor SS Timur HT Okyay RE Idiman E. Safe and successful use of oocyte in-vitro maturation in two infertile women with multiple sclerosis. Reprod Biomed Online. (2020) 41:154–6. doi: 10.1016/j.rbmo.2020.02.009 32536541

[120] Dobson R Dassan P Roberts M Giovannoni G Nelson-Piercy C Brex PA. UK consensus on pregnancy in multiple sclerosis: 'Association of British Neurologists' guidelines. Pract Neurol. (2019) 19:106–14. doi: 10.1136/practneurol-2018-002060 30612100

[121] Liguori NF Alonso R Pinheiro AA Balbuena ME Barboza A Bestoso S. Consensus recommendations for family planning and pregnancy in multiple sclerosis in Argentina. Mult Scler Relat Disord. (2020) 43:102147. doi: 10.1016/j.msard.2020.102147, PMID: 32442883

[122] Batista S Da Silva AM Sá MJ Sousa L De Sá J Pedrosa R. Recommendations about multiple sclerosis management during pregnancy, partum and post-partum: consensus position of the portuguese multiple sclerosis study group. Acta Medica Port. (2020) 33:611–21. doi: 10.20344/amp.12777, PMID: 32893778

[123] Oreja-Guevara C Rabanal A Rodríguez CH Benito YA Bilbao MM Gónzalez-Suarez I. Assisted reproductive techniques in multiple sclerosis: recommendations from an expert panel. Neurol Ther. (2023) 12:427–39. doi: 10.1007/s40120-023-00439-y, PMID: 36746871 PMC10043068

