# Supplementary material for: Optimizing the “Time to pregnancy” in women with multiple sclerosis: the OPTIMUS Delphi survey
Source: Front Neurol. 2023 Oct 6;14:1255496. doi: 10.3389/fneur.2023.1255496 (PMC10588727; doi:10.3389/fneur.2023.1255496)
Supplement: Supplementary file 1 [file Table_1.DOCX]

Supplementary Material

**OPTIMIZING THE “TIME TO PREGNANCY” IN WOMEN WITH MULTIPLE SCLEROSIS: THE OPTIMUS DELPHI SURVEY.**

**Luigi Carbone^1#*^, Doriana Landi^2#^, Raffaella Di Girolamo^3^, Paola Anserini^4^, Diego Centonze^5^, Girolama Alessandra Marfia^2§^, Carlo Alviggi^3§^, on behalf of the Interdisciplinary Group for Fertility in Multiple Sclerosis (IGFMS)^**

*** Correspondence:** Corresponding Author: drcarboneluigi@gmail.com

# Supplementary Table

| **Statement** | **Comments** |
| --- | --- |
| 1. | Particularly in MS women with age > 30-35 years. |
|  | It depends on whether the woman wants children in the future, whether she already has them, on her age and sexual orientation. |
| 3. | Consider potential obstacles to pregnancy/motherhood due to severe disability. |
|  | It depends on age and treatment options/timing. |
| 4. | In women who wish to have children only. |
| 5. | Interpretation, should be made by gynaecologists and made available to neurologists. This information can be used by neurologists during counselling. |
| 6. | "All women" encompasses menopausal women or women who have already had children. |
| 7. | "All men" encompasses men who have already had children, do not want them or are homosexual. |
| 10. | Consider disease activity. |
|  | Only if MS is not aggressive. |
|  | Not if patients are on second-line DMDs. |
|  | Also, glatiramer acetate, not only IFNs. |
|  | It is the best option available. |
|  | Yes, if patient is in treatment with first line DMT. |
| 11. | Wash out duration should be also limited, when possible, considering for instance "bridging therapies". |
| 12. | More scientific evidence is needed. |
|  | More studies are warranted to explore this specific issue. |
|  | Lymphocyte count must be provided. |
|  | It depends on whether severe lymphopenia is present. |
| 12. revote | It could be safe, but being described so in the Summary of Product Characteristics, it would configure an off-label practice |
|  | Further evidence is strongly advisable to confirm the statement and indicate an acceptable wash-out period |
|  | More evidence is needed. |
|  | My personal agreement has to be considered in the perspective of ascertaining this possibility by mean of a properly designed study |
|  | Lymphopenia may be long-lasting. Evidence of its effect on the embryogenesis and on the newborn health is lacking. |
|  | Although it is reasonable, data from pregnancy registries is desirable |
|  | Based on elimination time I think it's possible |
| 13. | A shared decision with information about the risk of disease reactivation is needed. |
|  | With a caveat for GnRH agonists. |
|  | Not totally, because of only few studies |
| 14. | Consider disease activity. |
|  | This item needs to be considered considering the fertility of the couple as a whole in the context of the fertility medical history. |
| 14. revote | ART should be reserved to patients in whom natural conception can't be pursued. |
|  | There is still the need for evidence-based data on this specific issue. |
|  | Cautions should be used if GnRH agonists are needed as stimulating therapy |
|  | The same opinion would apply to women without MS |
| 16. | Depends on the technique |
| 17. | Prioritize optimization of therapy and disease control before pregnancy planning. |
|  | This cannot be considered a rule or even a indication in expert opinions or guidelines, since the definition of suboptimal is not unequivocal and could probably depend on disease history and the planned therapy, and also because "time to pregnancy" could not be easily planned. |
|  | It depends on the age and on the possibility of switching for example to natalizumab. |
| 18. | Does this mean that a therapy should be switched for a reason different from disease activity or response to current treatment? I find it difficult to justify it in front of regulatory agencies. |
|  | I have no experience in this field, but I think I would switch to a high-efficacy DMD only in case of proven increased MS activity. |
|  | Depends on the disease course/activity over the previous years. |
|  | It depends on MS course and clinical/neuroradiological activity. |
|  | Desire of pregnancy must be always weighted with MS prognosis. This must be discussed in advance. |
|  | With natalizumab. |
| 18. revote | High-efficacy DMD potentially compatible with pregnancy. |
|  | The switch needs to be driven by the clinical characteristics and can't be done in a stable patient only because more than one ART might be required. |
|  | It depends on disease history; the sentence in the way is proposed is not unequivocal. |
|  | Disease activity would be the main driver for treatment choices in any case. |
| 20. | Are there any second-line DMD licensed for use during pregnancy? |
| 21. | In this case, I prefer to postpone ART in the long term. |
| 22. | I have no experience in oocyte cryopreservation, since none of my patients has needed it. In clinical practice the time to achieve the diagnosis is quite short; many available MS drugs are compatible with pregnancy granting a good disease control. |
|  | It is not acceptable a long period to achieve diagnosis; semestral or annual monitoring is demanded and sufficient to make better decisions. |
| 23. | Depending on age. |
|  | It depends on the age. |
| 24. | Yes, it is not a routine procedure in MS centers. |
|  | Financially difficult to support. |
